# Synthesis of trimetallic oxide (Fe_2_O_3_–MgO–CuO) nanocomposites and evaluation of their structural and optical properties

**DOI:** 10.1038/s41598-023-39845-5

**Published:** 2023-08-09

**Authors:** A. H. Al-Hammadi, Adnan Alnehia, Annas Al-Sharabi, Hisham Alnahari, Abdel-Basit Al-Odayni

**Affiliations:** 1 Department of Physics, Faculty of Sciences, Sana’a University, Sana’a, 12081 Yemen; 2 Department of Physics, Faculty of Applied Sciences, Thamar University, Dhamar, 87246 Yemen; 3 Engineer Abdullah Bugshan Research Chair for Dental and Oral Rehabilitation, College of Dentistry, King Saud University, 11545 Riyadh, Saudi Arabia

**Keywords:** Materials science, Nanoscience and technology

## Abstract

In this paper, tri-phase Fe_2_O_3_–MgO–CuO nanocomposites (NCs) and pure CuO, Fe_2_O_3_ and MgO nanoparticles (NPs) were prepared using sol–gel technique. The physical properties of the prepared products were examined using SEM, XRD, and UV–visible. The XRD data indicated the formation of pure CuO, Fe_2_O_3_ and MgO NPs, as well as nanocomposite formation with Fe_2_O_3_ (cubic), MgO (cubic), and CuO (monoclinic). The crystallite size of all the prepared samples was calculated via Scherrer's formula. The energy bandgap of CuO, Fe_2_O_3_ and MgO and Fe_2_O_3_–MgO–CuO NCs were computed from UV–visible spectroscopy as following 2.13, 2.29, 5.43 and 2.96 eV, respectively. The results showed that Fe_2_O_3_–MgO–CuO NCs is an alternative material for a wide range of applications as optoelectronics devices due to their outstanding properties.

## Introduction

Due to their unique optical, electrical, thermal, photocatalytic, mechanical, adsorbent and structural properties, metal oxide (MO) nanocomposites (NCs) have attracted much attention in recent years^1–5^. The NCs are composed of two or more nano-oxides and possessing properties which depend on the concentration of each constituent oxide in the mixture^6–8^. They are useful in a variety of applications, including solar cells, photovoltaic instruments, battery materials, gas sensors, and fuel cells^9–15^. Copper oxide (CuO) is a p-type semiconductor with a narrow bandgap of 1.2 eV^8^. It has unique optical and structural properties with low-cost preparation. It has attracted considerable attention due to its potential applications in superconductivity, gas sensing, solar cell and supercapacitor^16,17^. Furthermore, it is a non-toxic and readily available semiconductor^18,19^. Magnesium oxide (MgO), with a direct bandgap of 5.2–7 eV, is an n-type semiconductor that displays noticeable structural, catalytic, optical, and chemical properties^17,20–22^. Iron(III) oxide (Fe_2_O_3_) is a narrow bandgap of nearly 2 eV. It is associated with certain features, like the low toxicity, low cost, magnetic behavior and high solubility^23,24^. Hence, it is engaged in various applications involving biomedicine, cosmetics, diagnostics, sensors, radiology, and vaccines^9,23,25,26^.

By combining the different metal oxides (MOs) to form new NCs, various properties of individual oxide could significantly enhanced and, consequently, open up a new avenue of research for optoelectronics, electrical, thermal, photo-catalysis, and biological applications^26,27^. Mixed metal oxide NCs can be fabricated via different approaches such as the co-precipitation^28^, sonochemical^7^, solution combustion^29^, microwave technique^10^, ultrasonic-assisted^30^ and green methods^2,11^.

In this work, tri-phase Fe_2_O_3_–MgO–CuO NCs and pure CuO, Fe_2_O_3_ and MgO NPs were prepared using sol–gel method. It has the advantages of being environmentally friendly, simple, cheap and fast to perform without any special equipment. Herein, the novelty lies in the designed combination of the three metal oxides in one NC, which supposedly could lead to enhanced properties and potential applications. The obtained oxides were characterized for their structural and optical properties using XRD, UV–visible, and SEM.

## Materials and methods

### Materials

Magnesium nitrate hexahydrate (Mg(NO_3_)_2_·6H_2_O; 97%), Iron nitrate nonahydrate (Fe(NO_3_)_3_·9H_2_O; 97%), copper nitrate trihydrate (Cu(NO_3_)_2_·3(H_2_O; 98%) and absolute ethanol were purchased from BDH and used as received without additional treatment.

### Synthesis

The sol–gel method^20,31^ was used to fabricate the Fe_2_O_3_–MgO–CuO NCs, which involves the following steps: Cu(NO_3_)_2_·3(H_2_O) (3.382 g in 20 mL ethanol), Fe(NO_3_)_3_·9(H_2_O) (5.65 g in 20 mL ethanol) and Mg(NO_3_)_2_·6(H_2_O) (3.589 g in 20 mL ethanol) with constant molar ratio (1:1:1) were synthesized as three separate solutions. Each solution was stirred for 10 min at 23 ± 2 °C to obtain a homogeneous solution. The solutions were mixed under constant stirring for 70 min at 80 °C until gel was obtained. After that, the gel burns to create xerogel, which grinded to fine powder and annealed at 800 °C for 90 min. The individual pure oxides (Fe_2_O_3_, CuO, and MgO) were separately prepared following similar steps as composite, using the corresponding salt.

### Instruments

The optical properties of the synthesized materials were investigated using UV–Vis spectrophotometer (Hitachi U3900 with a software of Varian Cary 50). The structural properties were investigated by X-ray diffraction (XRD) using a Shimadzu EDX-720 (China) with CuKα radiation (λ = 0.154 nm). Morphological properties were assessed using SEM machine from JEOL (Jeol Ltd., Tokyo, Japan).

## Results and discussion

The structural integrity of the synthesized metal oxides is confirmed via powder X-ray crystallography. The targeted substances were obtained via sol–gel route followed by calcination at 800 °C. The annealing temperature of 800 °C suggests high crystalline products as reported elsewhere^32^. However, such high temperature could stimulate production of pure substances with better performance.

### X-ray diffraction

**Figure 1:**
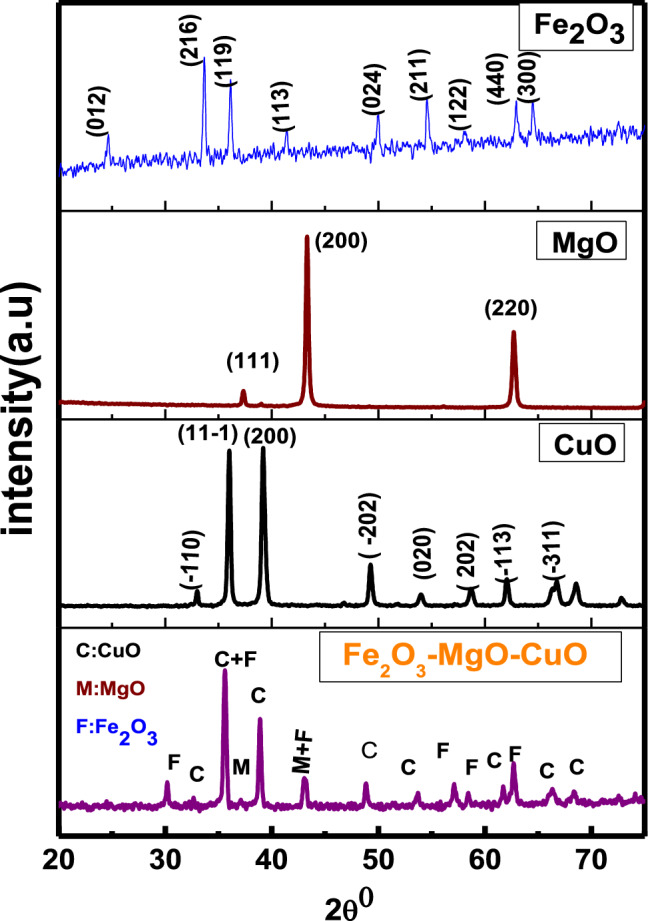
XRD patterns of CuO, Fe_2_O_3_, MgO and Fe_2_O_3_–MgO–CuO nanocomposites.

The crystalline arrangements and phase of the prepared nanopowder are estimated by XRD. Figure 1 shows the XRD pattern of the fabricated Fe_2_O_3_–MgO–CuO NCs. The observed diffraction peaks of pure oxides are close to the diffraction patterns reported in the X-ray database of JCPDS CuO (45-0937), Fe_2_O_3_ (33-0664) and MgO (45-0946). Similarly, in Fe_2_O_3_–MgO–CuO NCs, the diffraction patterns of CuO (48-1548), Fe_2_O_3_ (39-1346), and MgO (45-0946) match well with their respective standard reference cards. The diffracted peaks in composite were assigned for MgO (cubic), Fe_2_O_3_ (cubic) and CuO (monoclinic) phases. The characteristic diffraction peaks of CuO, Fe_2_O_3_ and MgO are well specified with no peaks relating to secondary or impurity segments or hydroxide in the sample, confirming the successful growth of Fe_2_O_3_–MgO–CuO NCs. The crystalline nature of the sample is assessed based on the sharp and strong diffraction peaks in Fig. 1. The cell volume (v), lattice constants (a, b, c) and d-spacing for pure CuO monoclinic, MgO cubic and Fe_2_O_3_ hexagonal phase and Fe_2_O_3_–MgO–CuO nanocomposite were calculated^22,33–36^ and listed in Table 1.

**Table 1:** Geometric parameters of CuO, Fe_2_O_3_ and MgO in grown Fe_2_O_3_–MgO–CuO nanocomposites determined from XRD analysis.

Material	Oxide	ID phase	a (Å)	b (Å)	c (Å)	Volume (Å^3^)	d-spacing (Å)
Pure oxides (CuO, Fe_2_O_3_, MgO)	CuO	Monoclinic	4.685	3.426	5.130	82.3	1.875
	Fe_2_O_3_	Hexagonal	5.036	5.036	13.749	301.9	1.966
	MgO	Cubic	4.211	4.211	4.211	74.7	1.992
Nanocomposite (CuO–Fe_2_O_3_–MgO)	CuO	Monoclinic	4.688	3.423	5.132	81.2	2.523
	Fe_2_O_3_	Cubic	8.351	8.351	8.351	582.4	2.521
	MgO	Cubic	4.211	4.211	4.211	74.7	2.099

The Scherrer equation^37^ was utilized to compute the crystallite size (D) of CuO, Fe_2_O_3_, MgO and Fe_2_O_3_–MgO–CuO NCs. Then, their dislocation density was also calculated^1,38,39^, Table 2. As can be seen, the average D values of CuO, and MgO were larger than in the NCs as compared with individual oxides, due to the agglomeration of particles caused by the presence of Fe_2_O_3_. The Fe_2_O_3_ particles act as nucleation sites for the CuO and MgO particles, resulting in aggregation into larger clusters. This phenomenon is known as the Ostwald ripening effect, where smaller particles dissolve and re-deposit on larger particles, resulting in an increase in their size. Hence, the presence of Fe_2_O_3_ in the NCs leads to an increase in the particle size of CuO and MgO.

**Table 2:** Structural parameters of CuO, Fe_2_O_3_ and MgO in grown Fe_2_O_3_–MgO–CuO nanocomposites determined from XRD analysis.

Material	Oxide	Average crystallite size (nm)	Average dislocation density (lines/m^2^) × 10^14^
Pure (CuO, Fe_2_O_3_, MgO)	CuO	22.110	20.456
	Fe_2_O_3_	89.141	1.258
	MgO	30.120	11.022
Nanocomposite (CuO–Fe_2_O_3_–MgO)	CuO	51.457	3.777
	Fe_2_O_3_	55.954	3.194
	MgO	60.305	2.749

### SEM analysis

**Figure 2:**
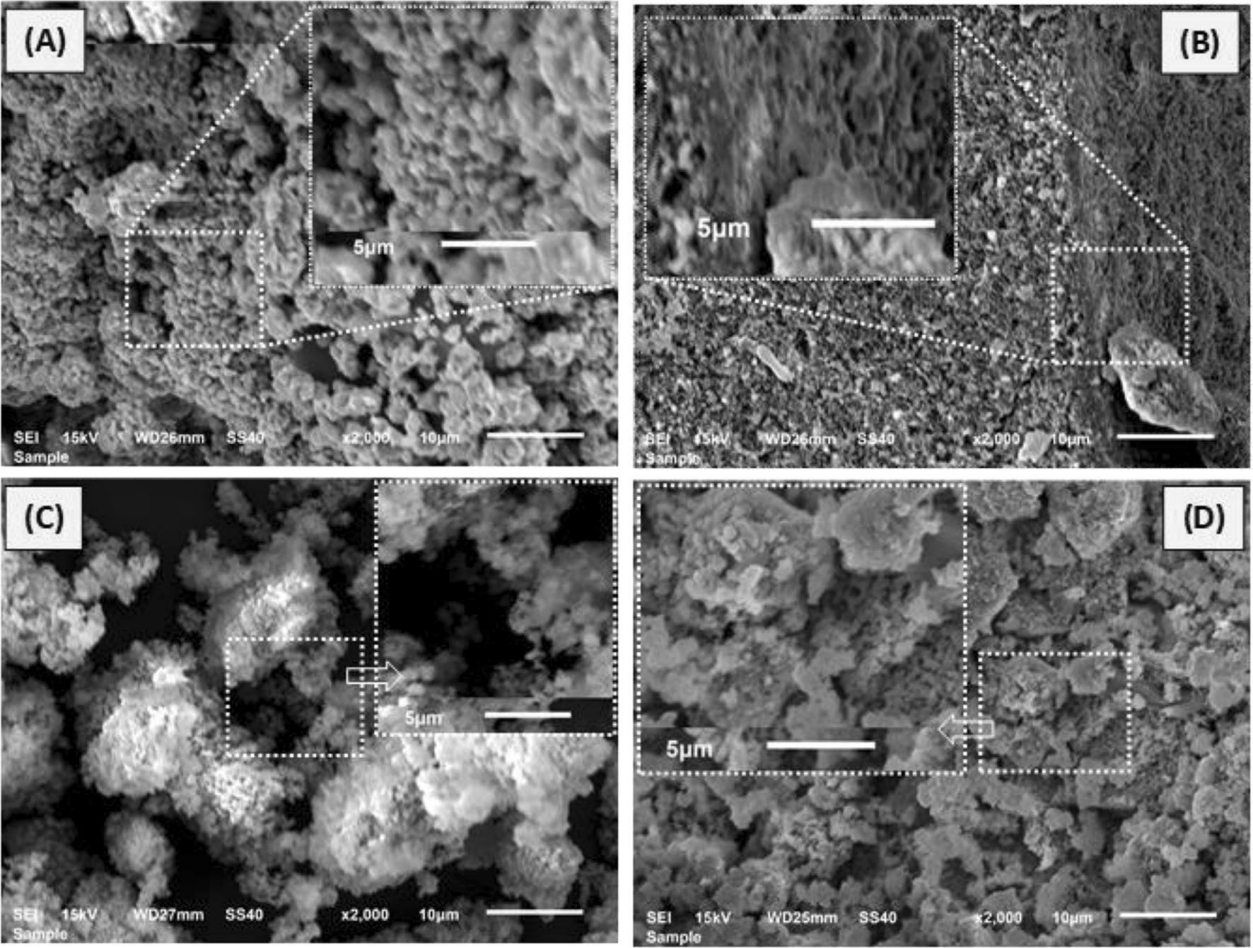
SEM images of (**A**) CuO, (**B**) Fe_2_O_3_, (**C**) MgO, (**D**) Fe_2_O_3_–MgO–CuO nanocomposites. Inserts are magnification of the shown selected area.

Figure 2 represents the SEM images of grown pure CuO, Fe_2_O_3_, MgO and Fe_2_O_3_–MgO–CuO NCs. It is seen that the formed nanostructures have spherical shapes with hardly distinct morphology. Furthermore, due to the low resolution of the presented SEM images, the non-size and thus, particle sizes and distribution are difficult to be counted. Nevertheless, the XRD data supported the claimed nanostructures. To improve the seen, and thus the suggested nanostructures, a higher magnification of the SEM image was presented as an insert within the corresponding image. The resulting magnified view is simply support that the particles are in nanometer range. In addition, some nanoparticles are well separated and thus could be counted. For example, by counting of the obviously countable particles of the composite image (Fig. 2D), it is found that the averaged particle size is 153 ± 30 nm, which is higher than that calculated from XRD (56 ± 4 nm) shown in Table 2. According to literature^40,41^, the SEM-based particle size is often larger than those measured by other techniques like XRD, the case that can be seen herein.

### UV–Vis spectroscopy

**Figure 3:**
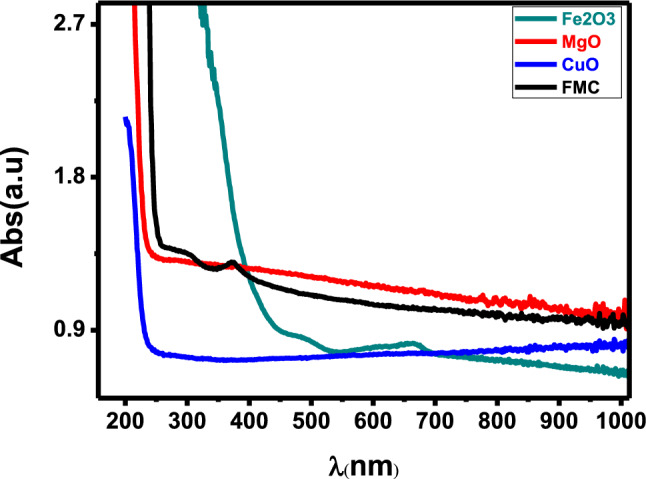
Absorption spectra of CuO, Fe_2_O_3_, MgO and Fe_2_O_3_–MgO–CuO (FMC) nanocomposites.

The optical properties of the Fe_2_O_3_–MgO–CuO NCs were studied by UV–visible spectroscopy. Figure 3 displays the absorption spectrum of Fe_2_O_3_–MgO–CuO NCs within 200–1000 nm. The absorption spectrum of the scattering radiation is observed in the longer wavelength region, and a larger-tail is seen due to the mixing of different oxides.

**Figure 4:**
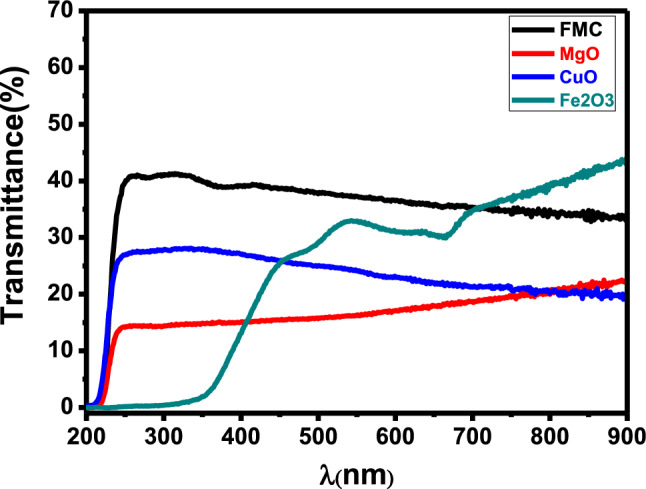
Transmission spectra of CuO, Fe_2_O_3_, MgO and Fe_2_O_3_–MgO–CuO (CFM) nanocomposites.

The transmission spectra of all the synthesized materials showed almost an opposite behavior to that seen in Fig. 4. Obviously, the optical transmission increased in the visible region for all the synthesized materials and possesses maximum value for Fe_2_O_3_–MgO–CuO NCs. The absorption coefficient (α) value can be computed via the following equation $\mathrm{\alpha }=\frac{2.303A}{t}$^34^.

**Figure 5:**
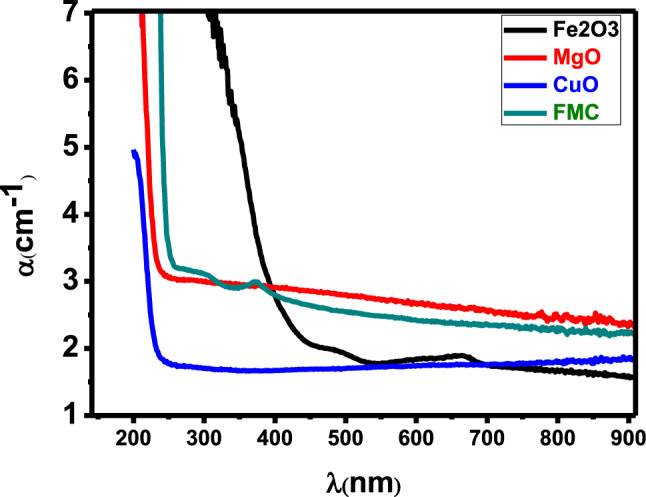
Absorption coefficient versus wavelength of CuO, Fe_2_O_3_, MgO and Fe_2_O_3_–MgO–CuO (FMC) nanocomposites.

The change in α (λ) for CuO, Fe_2_O_3_, MgO and Fe_2_O_3_–MgO–CuO NCs is presented in Fig. 5. From this Figure, it can be seen that α decreases as the wavelength ($\lambda$) of the incident photon increases. The extinction coefficient (α) value can be calculated via the following equation $k=\frac{\mathrm{\alpha }\lambda }{4\pi }$^42,43^.

**Figure 6:**
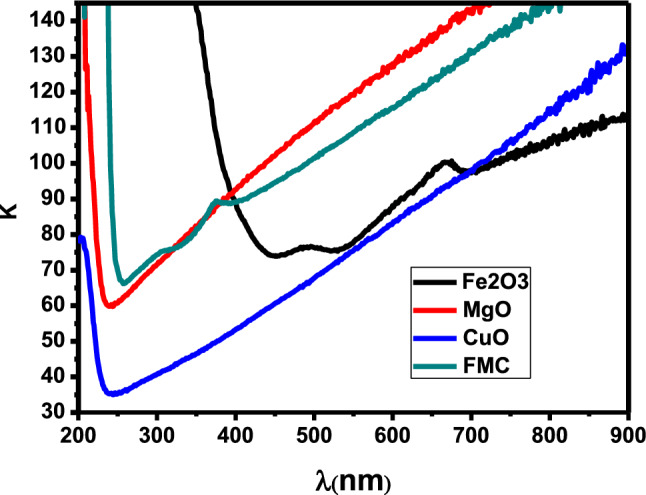
Extinction coefficient (k) versus wavelength of CuO, Fe_2_O_3_, MgO and Fe_2_O_3_–MgO–CuO (FMC) nanocomposites.

The change in $k$ (λ) for CuO, Fe_2_O_3_, MgO and Fe_2_O_3_–MgO–CuO NCs is presented in Fig. 6. It can be observed that $k$ increases as the wavelength of the incident photon increases.

**Figure 7:**
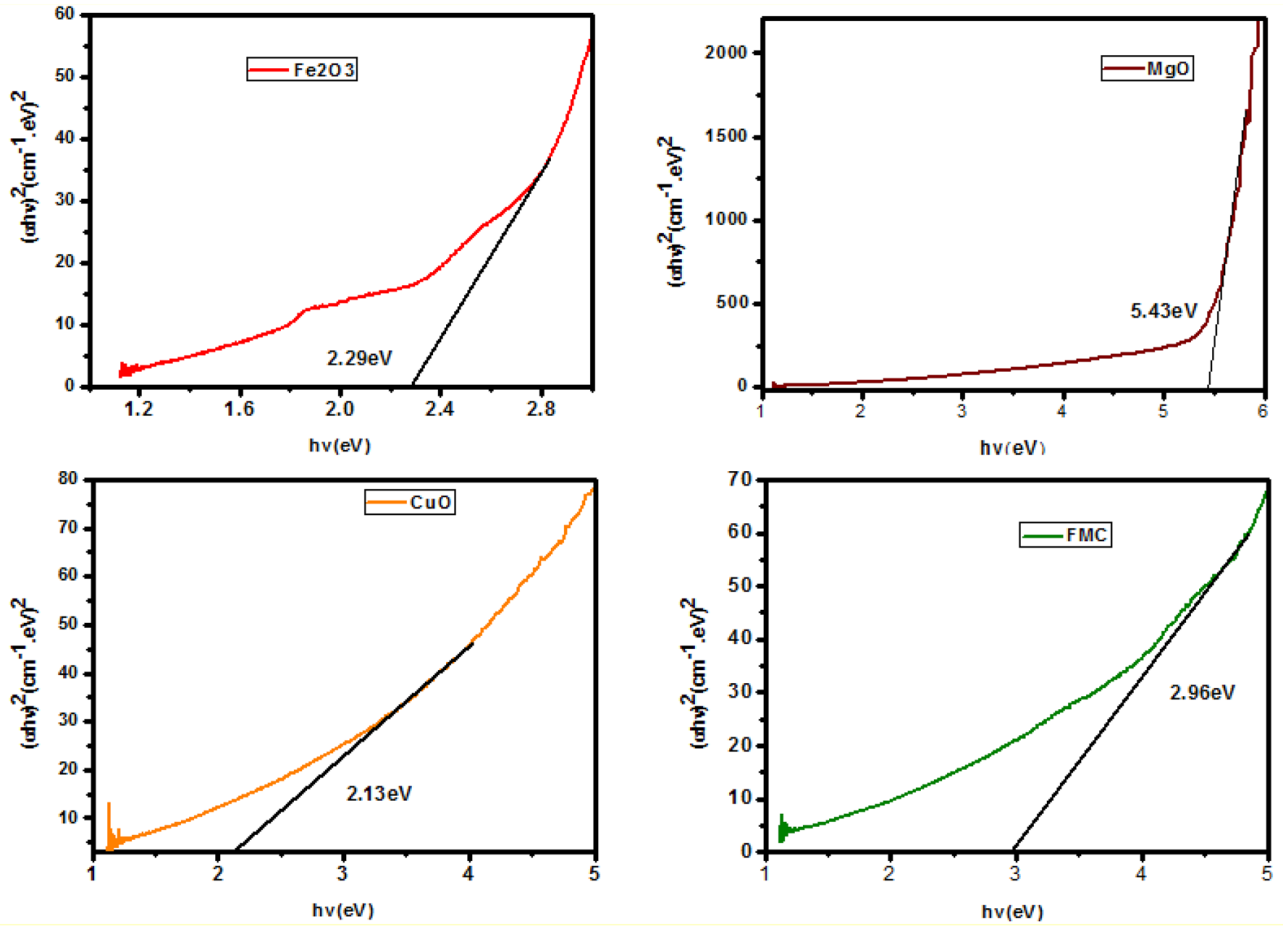
Optical bandgap plots for CuO, Fe_2_O_3_, MgO and CuO–Fe_2_O_3_–MgO (FMC) nanocomposite.

The energy bandgap (Eg) values of CuO, Fe_2_O_3_, MgO and Fe_2_O_3_–MgO–CuO NCs for the direct electronic transition between the valence band (VB) and conduction band (CB) can be computed via Tauc's relation^38,44,45^ as shown in Fig. 7. The ${E}_{g}$ values of CuO, Fe_2_O_3_, MgO NPs were calculated to about 2.13, 2.29, and 5.43 eV, respectively. However, Fe_2_O_3_–MgO–CuO NCs displayed the ${E}_{g}$ of 2.96 eV. In comparison to individual CuO, Fe_2_O_3_, MgO NPs, Fe_2_O_3_–MgO–CuO displayed significant increased absorbance in the visible region due to incorporation of three metal oxide. The reason for the change in the bandgap energy of the NCs compared to the individual metal oxides is likely due to the formation of new energy states at the interfaces between the different metal oxides. This can result in a shift in the electronic structure and a change in the bandgap energy. Additionally, the presence of multiple metal oxides in the NCs can also lead to increased electron–hole separation and improved charge transport properties, which can further affect the bandgap energy. This result was in good agreement with the literature^46^, which showed the possibility of using the prepared materials in some optical application.

## Conclusion

In Summary, tri-phase Fe_2_O_3_–MgO–CuO NCs and pure CuO, Fe_2_O_3_ and MgO NPs were successfully fabricated using a sol–gel approach. The XRD emphasized the formation of pure CuO, Fe_2_O_3_ and MgO NPs and CuO–Fe_2_O_3_–MgO NCs. The variation in the average crystallite size (D) and lattice constant were observed due to the interaction of the corresponding metal oxides. The optical bandgap was reached 2.13, 5.43, 2.29 and 2.96 eV for CuO, MgO, Fe_2_O_3_ and Fe_2_O_3_–MgO–CuO NCs, respectively.

## Data Availability

The datasets generated and/or analyzed during the current study are available from the corresponding author on reasonable request.
